# Suicide, other externally caused injuries, and cardiovascular disease within 2 years after cancer diagnosis: A nationwide population‐based study in Japan (J‐SUPPORT 1902)

**DOI:** 10.1002/cam4.5122

**Published:** 2022-08-08

**Authors:** Ken Kurisu, Maiko Fujimori, Saki Harashima, Tatsuo Akechi, Tomohiro Matsuda, Kumiko Saika, Kazuhiro Yoshiuchi, Isao Miyashiro, Yosuke Uchitomi

**Affiliations:** ^1^ Division of Supportive Care, Survivorship and Translational Research, Group for Supportive Care and Survivorship Research, Institute for Cancer Control National Cancer Center Japan Tokyo Japan; ^2^ Department of Stress Sciences and Psychosomatic Medicine, Graduate School of Medicine The University of Tokyo Tokyo Japan; ^3^ Department of Psychiatry and Cognitive‐Behavioral Medicine Nagoya City University, Graduate School of Medical Sciences Nagoya Japan; ^4^ Division of International Health Policy Research, Institute for Cancer Control National Cancer Center Japan Tokyo Japan; ^5^ Cancer Control Center Osaka International Cancer Institute Osaka Japan; ^6^ Innovation Center for Supportive, Palliative and Psychosocial Care National Cancer Center Tokyo Japan

**Keywords:** cancer, cardiovascular disease, externally caused injuries, psychological support, suicide

## Abstract

**Background:**

This study aimed to investigate the risk of death by suicide, other externally caused injuries (ECIs), or cardiovascular disease for patients with cancer.

**Methods:**

We used data from the National Cancer Registry, which include the entire population in Japan. Patients diagnosed with cancer from January 1 to December 31, 2016 were included, and their follow‐up period was set to 2 years. The standardized mortality ratio (SMR) of death by suicide, other ECIs, and cardiovascular disease was calculated compared with the general population. Multivariate Poisson or negative binomial regression analysis was used to quantify the adjusted relative risks of factors of interest.

**Results:**

We evaluated 1,070,876 patients with cancer. The 2‐year follow‐up SMR was 1.84 (95% confidence interval [CI]: 1.71–1.99) for suicide, 1.30 (95% CI: 1.24–1.37) for other ECIs, and 1.19 (95% CI: 1.17–1.21) for cardiovascular disease. The SMR was higher with shorter follow‐up periods but was significant 13–24 months after cancer diagnosis. The SMRs at 0–1 month and 13–24 months, respectively, were 4.40 (95% CI: 3.51–5.44) and 1.31 (95% CI: 1.14–1.50) for suicide; 2.27 (95% CI: 1.94–2.63) and 1.27 (95% CI: 1.18–1.37) for other ECIs; and 2.38 (95% CI: 2.27–2.50) and 1.07 (95% CI: 1.04–1.10) for cardiovascular disease. The multivariate analyses showed that patients with cancers other than localized tumors had significantly high relative risks of death for each cause.

**Conclusion:**

Suicide prevention countermeasures for patients with cancer, especially those with advanced disease immediately after diagnosis, are warranted.

## Graphical abstract

The SMRs of death by suicide, other external caused injury, and cardiovascular disease, quantified using the National Cancer Registry in Japan, were higher in patients with cancer than in the general population. The SMRs were higher with shorter follow–up periods after cancer diagnosis.

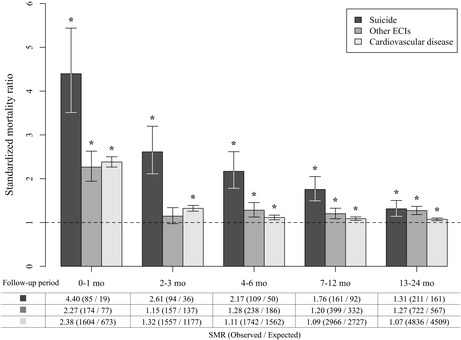

## 1 INTRODUCTION

Patients with cancer experience psychological distress throughout diagnosis and treatment, and mental comorbidities are common.^1^ Psychological distress reportedly increases the risk of suicide in patients with cancer.^2^, ^3^, ^4^, ^5^, ^6^ Such distress also increases the mortality rate due to other externally caused injuries (ECIs) in them,^7^ which may sometimes be a suicide misclassification.^8^, ^9^ Patients with cancer also have a high risk of death from cardiovascular disease,^2^, ^10^, ^11^, ^12^, ^13^ seemingly due to several factors, including psychological distress.^2^, ^12^, ^13^

Although these findings suggest a need for psychological support for patients with cancer, no nationwide study in Japan had investigated the risk of death from suicide, other ECIs, or cardiovascular disease among such patients. In this context, we previously investigated the risk of death from these causes within 6 months after cancer diagnosis using the National Cancer Registry (NCR).^14^ The study revealed a higher risk in patients with cancer than that in the general population. However, further investigation with a longer follow‐up period was warranted to explore preventive strategies. Multivariate analysis to determine high‐risk populations was also lacking from the previous study.

Since that study was conducted, more data have accumulated in the NCR database, and analysis with a 2‐year follow‐up and multivariate analysis became possible. Thus, the present study aimed to investigate the risk of death by suicide, other ECIs and cardiovascular disease within 2 years after cancer diagnosis.

## 2 MATERIALS AND METHODS

### 2.1 Study participants

The study protocol has been described elsewhere.^15^ We generally followed this protocol, but made some revisions, such as for the multivariate analyses. This population‐based study used the data of patients with cancer registered in the NCR, which covers the entire population of Japan. The NCR was launched in 2016 based on the Act on Promotion of Cancer Registries.^16^ From this database, we extracted the data of patients diagnosed with cancers between January 1 and December 31, 2016. The follow‐up period was set to 2 years from the cancer diagnosis.

The database records all cancer diagnoses notified since it was launched. We selected the earliest diagnosis date as the start of follow‐up for patients with multiple diagnostic records. This was because the earliest date was temporally closest to the date of first cancer diagnosis, shortly after which the risk of suicide rapidly increases, as shown in a previous study.^14^ Patients whose earliest diagnostic record in the NCR was that of a second or subsequent cancer were treated as patients with multiple primary tumors in the analysis.

The exclusion criteria were patients diagnosed through autopsy or by death certificate, those whose address was outside of Japan, and those with unknown age, sex, address, or time of diagnosis.

### 2.2 Outcome

The study outcome was death by suicide, other ECIs, or cardiovascular disease occurring during the follow‐up period. We used the following International Classification of Diseases, 10th Edition (ICD‐10) codes: suicide (X60–X84 and Y87.0), other ECIs (V01–X59, and Y10–Y34), and cardiovascular disease (I00–I99). Other ECIs included accidental causes such as car accidents, falls, and assaults.

### 2.3 Variables

We examined the following variables: age, sex, presence or absence of multiple primary tumors, primary tumor site, and extent of tumor (localized, regional, metastatic, and unknown/other).

The primary tumor site was categorized according to the ICD‐10 codes (Table 1). Patients with central nervous system (CNS) tumors, including benign diseases (ICD‐10 codes C70–C72, D320–D339, and D420–D439), were excluded from the analyses for cardiovascular disease due to potential misdiagnosis between CNS tumors and stroke.^2^

**TABLE 1:** Primary tumor site according to the ICD‐10 codes

Tumor site	ICD‐10 codes
Head and neck	C00–C14, C32
Esophagus	C15, D001
Stomach	C16
Colon	C18, D010
Rectum	C19–C20, D011–D012
Liver and intrahepatic bile ducts	C22
Gallbladder and other biliary tract	C23–C24
Pancreas	C25
Lung and bronchus	C33–C34, D021–D022
Skin	C43–C44, D030–D049
Breast	C50, D05
Cervix uteri	C53, D06
Corpus uteri	C54
Ovary	C56
Prostate	C61
Bladder	C67, D090
Kidney and urinary organs	C64–C66, C68
Brain and other parts of the central nervous system (CNS)^a^	C70–C72
Thyroid	C73
Malignant lymphoma	C81–C85, C96
Multiple myeloma	C88–C90
Leukemia	C91–C95
Others^a^	C00–C96 and D00–D48 not in the above

^a^ Patients with CNS tumors (C70–C72, D320–D339, and D420–D439) were excluded from the analysis of cardiovascular disease.

### 2.4 Statistical analysis

We quantified the standardized mortality ratio (SMR) adjusted by age and sex for each outcome, compared with the general Japanese population. SMR was calculated as the ratio of the observed death count for each cause among patients with cancer divided by the expected death count among the general population. The observed death counts were obtained from the NCR. The expected count was calculated as the sum of multiplying the follow‐up period of each patient by the cause‐specific mortality rate among the general population of the corresponding age, sex, and survival year in Japan. This cause‐specific mortality rate was derived from the death count by each cause (based on the Vital Statics Japan by the Ministry of Health, Labour and Welfare) and the total population (based on the Population Estimates). Patients registered with 0 survival months were assigned a value of 0.5 months. We used Byar's method to estimate the 95% confidence intervals (CIs) for the SMRs.

We performed multivariate Poisson or negative binomial regression analysis to evaluate the simultaneous effect of the variables of interest.^2^, ^3^ All variables of interest were included in the multivariate model. Negative binomial regression was employed when overdispersion was observed in the Poisson regression model through a dispersion test.^17^ To quantify the relative risks (RRs) for SMRs, we added an offset for a log of the expected number of deaths.^3^ We converted the NCR dataset, which includes individual data with a binary outcome, into a clustered dataset compiled by each variable, in which each cluster has count data as the outcome.^18^

To verify the robustness of the results from these analyses, we also performed a subgroup analysis excluding patients with multiple primary tumors.

All analyses were conducted using R (version 4.1.1) and the “epiR” (version 2.0.38), “MASS” (version 7.3–54), and “AER” (version 1.2–9) packages. *p*‐values <0.05 were considered to be statistically significant.

## 3 RESULTS

### 3.1 Patient characteristics

Table 2 presents the SMRs, observed death count, and expected death count of study participants. The analysis included 1,070,876 patients (1,049,901 for the analysis of cardiovascular disease after excluding patients with CNS tumors). This was obtained after excluding patients diagnosed through autopsy (*N* = 591), those diagnosed by death certificate (*N* = 33,797), those whose address was outside Japan (*N* = 257) or unknown (*N* = 402), and those with unknown age (*N* = 6), sex (*N* = 41), and time of diagnosis (*N* = 130).

**TABLE 2:** Standardized mortality ratio, observed count, and expected count of deaths by suicide, other externally caused injuries, and cardiovascular disease within the 2‐year follow‐up period

	No. of patients	SMR (95% CI) [Observed count/Expected count^a^]		
		Suicide	Other ECIs	CVD^b^
All	1,070,876	1.84 (1.71–1.99) [660/358]	1.30 (1.24–1.37) [1690/1298]	1.19 (1.17–1.21) [12,705/10,649]
Age (years)				
0–39	40,057	1.63 (0.91–2.69) [15/9]	0.64 (0.07–2.32) [N.A.]^c^	4.61 (2.69–7.39) [17/4]
40–49	68,834	2.36 (1.72–3.16) [45/19]	1.44 (0.74–2.51) [N.A.]^c^	2.22 (1.72–2.83) [65/29]
50–59	107,058	1.68 (1.31–2.12) [69/41]	1.92 (1.44–2.51) [53/28]	1.81 (1.59–2.06) [236/130]
60–69	265,556	1.96 (1.68–2.27) [178/91]	1.71 (1.50–1.94) [240/140]	1.59 (1.51–1.68) [1382/870]
70–79	321,616	1.98 (1.73–2.25) [230/116]	1.47 (1.35–1.60) [540/366]	1.35 (1.31–1.40) [3406/2515]
≥80	267,755	1.51 (1.26–1.80) [123/81]	1.12 (1.05–1.20) [843/753]	1.07 (1.05–1.09) [7599/7100]
Sex				
Female	479,922	1.99 (1.72–2.30) [187/94]	1.37 (1.25–1.49) [505/369]	1.22 (1.19–1.26) [4554/3719]
Male	590,954	1.79 (1.63–1.96) [473/264]	1.28 (1.20–1.35) [1185/929]	1.18 (1.15–1.20) [8151/6930]
Multiple primary tumors				
Present	137,262	1.57 (1.25–1.94) [84/53]	1.14 (1.00–1.29) [241/212]	0.83 (0.79–0.88) [1412/1696]
Absent	933,614	1.89 (1.74–2.05) [576/304]	1.33 (1.27–1.40) [1449/1086]	1.26 (1.24–1.28) [11,293/8952]
Primary tumor site				
Head and neck	24,794	2.35 (1.49–3.53) [23/10]	1.68 (1.26–2.20) [52/31]	1.13 (1.00–1.27) [280/249]
Esophagus	25,198	3.42 (2.34–4.83) [32/9]	1.39 (1.00–1.89) [41/29]	1.34 (1.20–1.50) [300/223]
Stomach	125,521	1.82 (1.45–2.25) [84/46]	1.39 (1.23–1.58) [258/185]	1.31 (1.25–1.37) [1992/1524]
Colon	128,329	1.63 (1.29–2.04) [77/47]	0.97 (0.83–1.13) [174/179]	1.15 (1.09–1.20) [1753/1528]
Rectum	58,882	1.92 (1.39–2.57) [44/23]	0.98 (0.76–1.25) [68/69]	1.16 (1.08–1.26) [648/557]
Liver and intrahepatic bile ducts	39,066	1.92 (1.20–2.90) [22/11]	1.86 (1.49–2.29) [88/47]	1.44 (1.33–1.57) [572/396]
Gallbladder and other biliary tract	20,858	2.23 (1.11–4.00) [11/5]	1.58 (1.12–2.16) [39/25]	1.21 (1.07–1.37) [271/223]
Pancreas	37,670	3.48 (2.23–5.17) [24/7]	1.79 (1.30–2.39) [45/25]	1.41 (1.25–1.58) [303/215]
Lung and bronchus	116,488	1.91 (1.47–2.44) [64/34]	1.47 (1.27–1.70) [187/127]	1.33 (1.26–1.41) [1384/1038]
Skin	30,171	1.37 (0.78–2.22) [16/12]	1.26 (1.01–1.54) [92/73]	1.10 (1.03–1.18) [785/711]
Breast	103,980	2.06 (1.52–2.74) [48/23]	0.95 (0.72–1.23) [58/61]	0.99 (0.91–1.08) [571/575]
Cervix uteri	33,045	1.97 (1.05–3.38) [13/7]	1.81 (0.93–3.15) [12/7]	1.30 (1.00–1.66) [65/50]
Corpus uteri	15,463	2.16 (0.87–4.45) [7/3]	1.23 (0.53–2.43) [N.A.]^c^	1.12 (0.86–1.44) [62/55]
Ovary	12,385	2.57 (0.94–5.59) [6/2]	0.90 (0.24–2.31) [N.A.]^c^	1.26 (0.93–1.68) [48/38]
Prostate	84,820	1.07 (0.79–1.42) [48/45]	1.00 (0.86–1.17) [174/173]	0.88 (0.83–0.93) [1150/1306]
Bladder	40,638	2.09 (1.48–2.87) [38/18]	0.98 (0.78–1.22) [80/81]	1.04 (0.97–1.12) [699/670]
Kidney and urinary organs	26,729	1.58 (0.90–2.56) [16/10]	1.12 (0.79–1.54) [38/34]	1.22 (1.09–1.36) [337/277]
Brain and other parts of the CNS	5915	1.31 (0.15–4.72) [N.A.]^c^	2.33 (1.00–4.60) [N.A.]^c^	Excluded^b^
Thyroid	17,285	0.79 (0.21–2.03) [N.A.]^c^	0.78 (0.34–1.55) [N.A.]^c^	0.91 (0.72–1.15) [74/81]
Malignant lymphoma	32,053	2.84 (1.90–4.08) [29/10]	1.04 (0.73–1.44) [36/35]	1.21 (1.08–1.34) [345/286]
Multiple myeloma	7037	2.28 (0.74–5.33) [N.A.]^c^	2.08 (1.25–3.25) [19/9]	1.89 (1.60–2.22) [150/79]
Leukemia	13,083	0.90 (0.18–2.64) [N.A.]^c^	3.67 (2.52–5.15) [33/9]	1.31 (1.06–1.61) [94/72]
Other	71,466	1.92 (1.39–2.58) [44/23]	2.29 (1.95–2.66) [168/74]	1.66 (1.55–1.78) [822/495]^b^
Extension of tumors				
Localized	543,101	1.38 (1.23–1.55) [295/213]	1.03 (0.96–1.11) [782/757]	0.96 (0.94–0.99) [5916/6144]
Regional	207,745	2.12 (1.79–2.49) [148/70]	1.38 (1.23–1.54) [329/239]	1.13 (1.09–1.18) [2222/1960]
Metastatic	166,891	3.28 (2.72–3.92) [120/37]	1.58 (1.37–1.81) [201/127]	1.42 (1.35–1.49) [1467/1035]
Unknown/other	153,139	2.52 (2.05–3.08) [97/38]	2.16 (1.94–2.38) [378/175]	2.05 (1.98–2.13) [3100 / 1509]

Abbreviations: CI, confidence interval; CNS, central nervous system; CVD, cardiovascular disease; ECI, externally caused injury; N.A., not available; SMR, standardized mortality ratio.

^a^ Expected count is shown in an integer form.

^b^ Patients with CNS tumors were excluded from the analyses.

^c^ N.A. is used when the observed count cannot be presented to anonymize the small number (<5) of events according to the National Cancer Registry privacy policy.

We observed 660 deaths caused by suicide, 1690 caused by other ECIs, and 12,705 caused by cardiovascular disease during the 2‐year follow‐up period. Among the 660 cases of death by suicide, 472 (72%) patients died at home.

### 3.2 Risk of death by suicide

**FIGURE 1:**
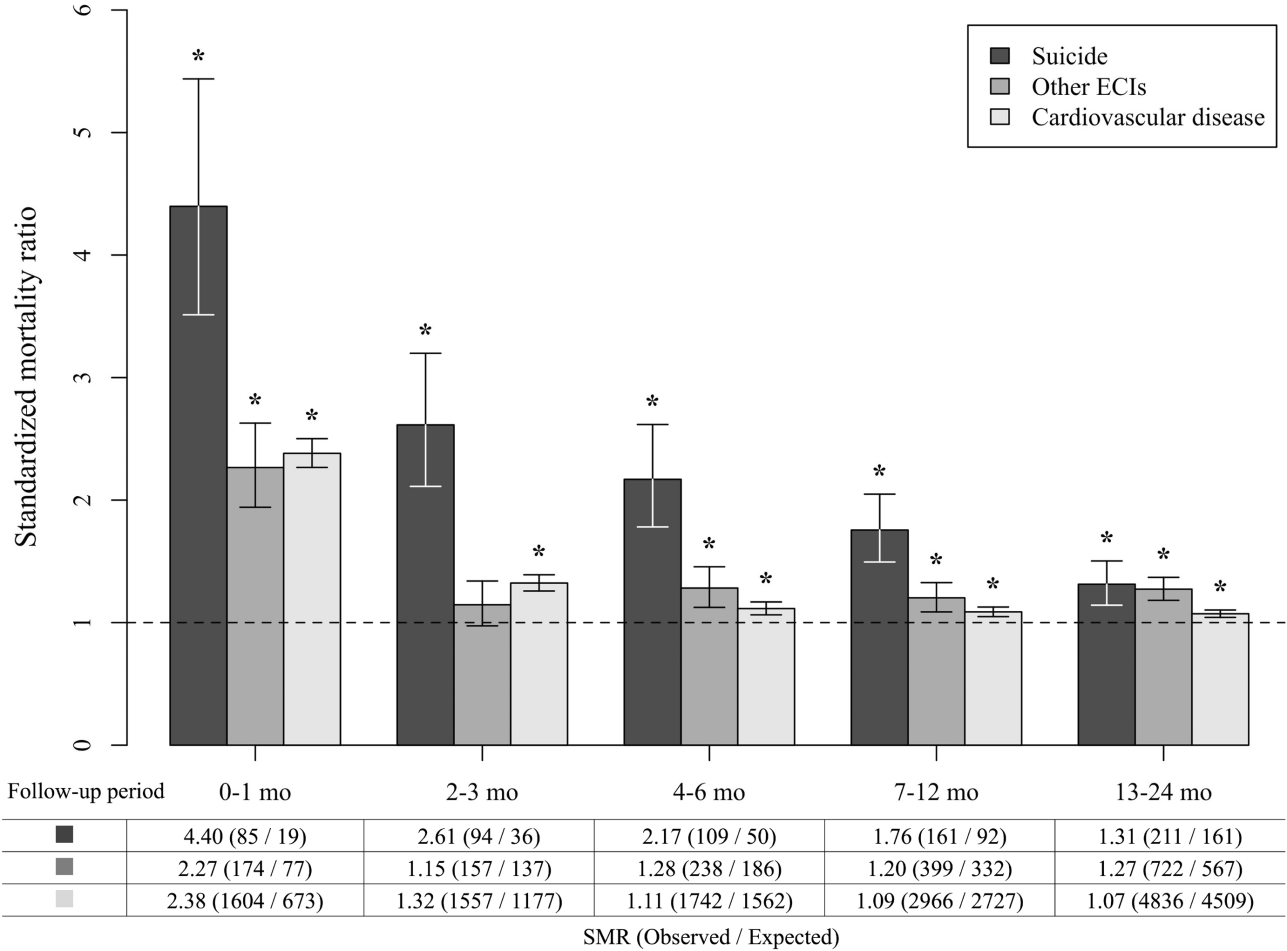
Standardized mortality ratio (SMR) by follow‐up period. * The SMR is significantly greater than 1.0 (*p* < 0.05).

The 2‐year follow‐up SMR for death by suicide was 1.84 (95% CI: 1.71–1.99) (Table 2). The SMR was higher with shorter follow‐up periods, decreasing from 4.40 (95% CI: 3.51–5.44) for 0–1 month to 1.31 (95% CI: 1.14–1.50) for 13–24 months (Figure 1).

The adjusted RRs of the multivariate Poisson regression model are given in Table 3. Patients with esophageal cancer and those with cancers other than localized tumors had high RRs of death by suicide. Those with prostate cancer had a relatively low RR. No significant differences were observed for age, sex, and presence of multiple primary tumors.

**TABLE 3:** Relative risk of death by suicide, other externally caused injuries, and cardiovascular disease within the 2‐year follow‐up period, adjusted for all factors of interest

	Relative risk (95% CI)		
	Suicide^a^	Other ECIs^a^	CVD^b^
Age (years)			
0–39	1.05 (0.58–1.87)	0.27 (0.07–1.11)	2.63 (1.59–4.34)*
40–49	1.44 (0.98–2.11)	0.73 (0.39–1.38)	1.28 (0.96–1.69)
50–59	1.00 (Reference)	1.00 (Reference)	1.00 (Reference)
60–69	1.21 (0.91–1.60)	0.91 (0.67–1.22)	0.94 (0.80–1.09)
70–79	1.26 (0.95–1.65)	0.77 (0.58–1.03)	0.77 (0.66–0.89)*
≥80	0.93 (0.69–1.25)	0.54 (0.41–0.72)*	0.50 (0.43–0.58)*
Sex			
Female	1.04 (0.85–1.29)	1.14 (1.01–1.28)*	1.15 (1.08–1.23)*
Male	1.00 (Reference)	1.00 (Reference)	1.00 (Reference)
Multiple primary tumors			
Present	0.84 (0.67–1.06)	0.90 (0.78–1.03)	0.70 (0.65–0.75)*
Absent	1.00 (Reference)	1.00 (Reference)	1.00 (Reference)
Primary tumor site			
Head and neck	1.40 (0.88–2.25)	1.65 (1.21–2.26)*	0.95 (0.80–1.13)
Esophagus	2.01 (1.33–3.04)*	1.36 (0.97–1.91)	1.13 (0.96–1.34)
Stomach	1.12 (0.82–1.53)	1.47 (1.21–1.78)*	1.14 (1.01–1.30)*
Colon	1.00 (Reference)	1.00 (Reference)	1.00 (Reference)
Rectum	1.11 (0.76–1.61)	0.95 (0.72–1.25)	0.93 (0.80–1.07)
Liver and intrahepatic bile ducts	1.21 (0.75–1.96)	1.92 (1.49–2.49)*	1.28 (1.10–1.49)*
Gallbladder and other biliary tract	1.14 (0.60–2.15)	1.46 (1.03–2.07)*	0.98 (0.82–1.17)
Pancreas	1.53 (0.96–2.44)	1.49 (1.07–2.08)*	1.04 (0.88–1.22)
Lung and bronchus	0.96 (0.69–1.34)	1.36 (1.11–1.68)*	1.07 (0.94–1.22)
Skin	1.03 (0.60–1.78)	1.58 (1.22–2.04)*	1.00 (0.85–1.19)
Breast	1.17 (0.79–1.75)	0.85 (0.63–1.16)	0.72 (0.61–0.85)*
Cervix uteri	1.23 (0.65–2.34)	1.62 (0.89–2.95)	0.84 (0.63–1.12)
Corpus uteri	1.29 (0.58–2.85)	1.06 (0.52–2.17)	0.81 (0.60–1.08)
Ovary	1.26 (0.54–2.96)	0.68 (0.25–1.85)	0.80 (0.59–1.10)
Prostate	0.62 (0.43–0.89)*	0.98 (0.79–1.21)	0.63 (0.54–0.74)*
Bladder	1.46 (0.98–2.16)	1.12 (0.86–1.46)	0.96 (0.82–1.12)
Kidney and urinary organs	0.95 (0.55–1.62)	1.09 (0.77–1.55)	0.99 (0.84–1.16)
Brain and other parts of the CNS	0.74 (0.18–3.04)	2.10 (1.03–4.28)*	Excluded
Thyroid	0.41 (0.15–1.13)	0.62 (0.31–1.27)	0.57 (0.44–0.74)*
Malignant lymphoma	1.22 (0.79–1.88)	0.87 (0.60–1.25)	0.85 (0.72–0.99)*
Multiple myeloma	0.85 (0.33–2.15)	1.28 (0.78–2.08)	0.81 (0.63–1.04)
Leukemia	0.33 (0.10–1.07)	2.19 (1.48–3.24)*	0.58 (0.44–0.75)*
Other	0.98 (0.67–1.44)	1.86 (1.49–2.32)*	1.01 (0.87–1.16)
Extension of tumors			
Localized	1.00 (Reference)	1.00 (Reference)	1.00 (Reference)
Regional	1.49 (1.21–1.83)*	1.35 (1.18–1.55)*	1.26 (1.16–1.36)*
Metastatic	2.37 (1.89–2.99)*	1.58 (1.34–1.86)*	1.57 (1.44–1.72)*
Unknown/other	2.09 (1.63–2.68)*	2.01 (1.75–2.31)*	2.43 (2.25–2.63)*

*Note*: All variables were included in the multivariate model simultaneously.

Abbreviations: CI, confidence interval; CNS, central nervous system; CVD, cardiovascular disease; ECI, externally caused injury; RR, relative risk; SMR, standardized mortality ratio.

^a^ Relative risk was quantified using Poisson regression model.

^b^ Relative risk was quantified using negative binomial regression model.

* *p* < 0.05.

### 3.3 Risk of death by other ECIs

The 2‐year follow‐up SMR for death by other ECIs was 1.30 (95% CI: 1.24–1.37) (Table 2). The SMR was higher with shorter follow‐up periods, from 2.27 (95% CI: 1.94–2.63) for 0–1 month to 1.27 (95% CI: 1.18–1.37) for 13–24 months (Figure 1).

The adjusted RRs of the multivariate Poisson regression model are given in Table 3. Patients with female sex, certain primary tumor sites (especially liver/intrahepatic bile ducts, CNS, and leukemia), and those with cancers other than localized tumors had high RRs of death by other ECIs. Those aged ≥80 years had a significantly low RR. No significant difference was observed for the presence/absence of multiple primary tumors.

### 3.4 Risk of death by cardiovascular disease

The 2‐year follow‐up SMR for death by cardiovascular disease was 1.19 (95% CI: 1.17–1.21) (Table 2). The SMR calculated for all patients (i.e., including those with CNS tumors) was 1.20 (95% CI: 1.18–1.22). The SMR was higher with shorter follow‐up periods, from 2.38 (95% CI: 2.27–2.50) for 0–1 month to 1.07 (95% CI: 1.04–1.10) for 13–24 months (Figure 1).

Due to significant overdispersion in the Poisson regression model detected by the dispersion test, we used a negative binomial regression model. The adjusted RRs of the model are given in Table 3. Patients with younger age, female sex, absence of multiple primary tumors, certain primary tumor sites (stomach and liver/intrahepatic bile ducts), and those with cancers other than localized tumors had high RRs of death by cardiovascular disease. Those with certain primary tumor sites, such as leukemia, had low RRs.

### 3.5 Analyses excluding patients with multiple primary tumors

The SMRs by follow‐up period and RRs from the multivariate analyses, quantified after excluding patients with multiple primary tumors, are shown in Figure S1 and Table S1, respectively. The temporal trends in the SMRs and the significance of variables for each outcome in the multivariate analyses were essentially the same as those found in the main analysis.

## 4 DISCUSSION

This population‐based study in Japan showed high SMRs of death by suicide, other ECIs, and cardiovascular disease in patients with cancer. These risks increased with shorter follow‐up periods after cancer diagnosis. The adjusted RRs were high in patients with cancers other than localized tumors for all causes of death. These results did not change after excluding patients with multiple primary tumors.

The high SMR of suicide, other ECIs, and cardiovascular disease in patients with cancer compared with those in the general population is consistent with results from studies in other countries.^2^, ^3^, ^4^, ^5^, ^6^, ^7^, ^10^, ^11^, ^12^, ^13^ We also observed higher SMRs for each cause of death in shorter follow‐up periods after diagnosis, consistent with reports from other countries.^2^, ^3^, ^5^, ^6^, ^7^, ^10^ Additionally, the SMR of suicide remained high even at 13–24 months after cancer diagnosis, whereas that of cardiovascular disease declined to the same level as that in the general population. These trends are also consistent with those of a previous study.^2^ Furthermore, the higher risk in patients with cancers other than localized tumors is consistent with previous reports, which suggest that patients with advanced cancer or poorer prognosis are at higher risk of death by each cause.^2^, ^5^, ^6^, ^7^

These results suggest the need for suicide prevention countermeasures, especially for patients with advanced cancer, immediately after diagnosis. In addition, suicide mainly occurred at home in Japan, which may require such countermeasures early at outpatient, as well as inpatient. The doctor–patient relationship is reportedly associated with the reduction of suicidal ideation in patients with cancer,^19^ and studies have been conducted to improve oncologists' communication skills.^20^ Further, to reduce the psychological distress of patients with cancer, several interventions have been examined, including improving unmet needs,^21^ screening and early consultation for mental symptoms,^22^ early palliative care,^23^ second opinion to reduce uncertainty,^24^ interventions to reduce cancer‐related stigma,^25^ and ketamine administration.^26^ Moreover, a study on the screening test of suicide risk showed an association between its completion and a lower rate of suicidal mortality.^27^ Application of such interventions to patients with advanced diseases immediately after diagnosis may be useful for investigating preventive strategies. In addition, other ECIs and cardiovascular disease in patients with cancer also appear to be related to psychological stress.^2^, ^7^, ^12^, ^13^ Thus, as with suicide prevention, psychological support would be important for preventing death due to these causes.

The RR of suicide in patients with esophageal cancer was high. This is consistent with the finding of a previous study, which noted the poor prognosis of esophageal cancer as a cause of the high risk of suicide.^3^ Surgery for esophageal cancer reportedly causes severe psychological distress.^28^ Alcohol and smoking are risk factors for suicide,^29^ and both are also associated with esophageal cancer,^30^ which could also explain the high RR of suicide with esophageal cancer. A study examining suicide among patients with esophageal cancer revealed several risk factors, such as histological grade.^31^ Further studies are warranted to investigate suicide in patients with esophageal cancer.

The analyses of other ECIs showed that the risk was higher for hepatocellular carcinoma, which is consistent with a previous study finding.^7^ These findings could be partially explained by alcohol being a risk factor for accidental death^32^ and sharing an association with hepatocellular carcinoma.^33^ Furthermore, the RRs for leukemia and CNS tumors were high, which were also shown in previous studies.^7^, ^34^ The reasons for these results may include cognitive dysfunction due to CNS tumors^7^ and neurological complications associated with hematopoietic stem cell transplantation.^34^ Further investigation of such an association between ECIs and neurological disturbance in patients with cancer is warranted. The RR was higher for female patients, which conflicts with a previous study from the United States.^7^ Such a difference between sex might vary between countries.

The analysis of cardiovascular disease showed several factors with significantly high RRs. First, the higher risk in younger patients is consistent with previous studies.^2^, ^10^, ^11^ This is likely because older patients generally have a high risk of cardiovascular disease, regardless of cancer,^35^ which could decrease the SMR adjusted by age. Second, the analysis showed that female patients had higher RRs than male patients; this has not been observed in previous studies from other countries. This finding might suggest the importance of considering sex differences in the psychological support of patients with cancer in Japan. However, the effect size was not substantial; thus, further validation of this finding is warranted. Third, the analyses showed a lower risk of cardiovascular death in patients with multiple primary tumors. Patients with multiple primary tumors, compared with those without, generally have a longer period since the initial cancer diagnosis. Thus, the lower risk of cardiovascular death could be explained by this longer period because the SMR decreased with a longer follow‐up period after diagnosis, as shown in Figure 1. Finally, certain primary tumor sites (gastric cancer and hepatocellular carcinoma) were found to be significant. However, the effect sizes were relatively small; thus, their clinical implication warrants further investigation.

This study had several limitations. First, we could not include potential risk factors for suicide and cardiovascular disease, such as alcohol use, obesity, and psychiatric comorbidities,^35^, ^36^ due to the use of the NCR. Second, the history of chemotherapy was not used in the analysis of cardiovascular disease for several reasons. The NCR only includes the history of chemotherapy administered for curative purposes. In addition, the types of chemotherapeutic agents administered were unclear, which prevented us from determining whether each patient had received cardiotoxic chemotherapy. Further, the history of chemotherapy can be updated after the initial registration, which may cause survivorship bias. Third, we did not conduct analyses stratified by each cancer type due to the limited sample size. A previous study suggested that patients with some types of cancer, such as testicular cancer, showed a higher risk of suicide with longer follow‐up,^4^ which might conflict with our results. Thus, to determine appropriate care, stratified analyses should be performed once sufficient data have accumulated in the NCR. Finally, the present study used data from 2016 to 2018 and set the maximum follow‐up term to 2 years. Further longitudinal surveillance is warranted to validate the present findings.

In conclusion, the SMRs of death by suicide, other ECIs, and cardiovascular disease were high in patients with cancer compared with those in the general population even 2 years after diagnosis. The risk of death due to these causes was especially high in patients with advanced disease, immediately after diagnosis. Suicide prevention countermeasures for such patients are warranted. However, longitudinal surveillance, analyses with a longer follow‐up period, and analyses stratified by cancer type should be performed once further data have accumulated.

## AUTHOR CONTRIBUTIONS

Ken Kurisu prepared the protocol, performed statistical analyses, authored, and revised the article. Maiko Fujimori supervised the study, prepared the protocol, and reviewed and edited the article. Saki Harashima, Tatsuo Akechi, Tomohiro Matsuda, Kumiko Saika, Kazuhiro Yoshiuchi, Isao Miyashiro, and Yosuke Uchitomi prepared the protocol, reviewed and edited the article.

## CONFLICT OF INTEREST

The authors indicated no potential conflicts of interest.

## ETHICAL APPROVAL STATEMENT

The study was approved by the institutional review boards of the National Cancer Center Japan (approval number: 2018–233). The requirement for informed consent was waived owing to the use of anonymous data.

## Supporting information


Figure S1
Table S1

## Data Availability

The dataset analyzed in this study is used with the permission of the National Cancer Registry Information Desk at the National Cancer Center. Therefore, this dataset cannot be shared, but anyone can use the dataset by submitting an application to the National Cancer Registry Information Desk under a study protocol.
