# Supplementary material for: Suicide, other externally caused injuries, and cardiovascular disease within 2 years after cancer diagnosis: A nationwide population‐based study in Japan (J‐SUPPORT 1902)
Source: Cancer Med. 2022 Aug 8;12(3):3442–51. doi: 10.1002/cam4.5122 (PMC9939211; doi:10.1002/cam4.5122)
Supplement: Supplementary file 1 — Figure S1 Table S1 [file CAM4-12-3442-s001.docx]

**Supplementary Figure 1. Standardized mortality ratio (SMR) by follow-up period, excluding patients with multiple primary tumors.**

* The SMR is significantly greater than 1.0 (p <0.05).


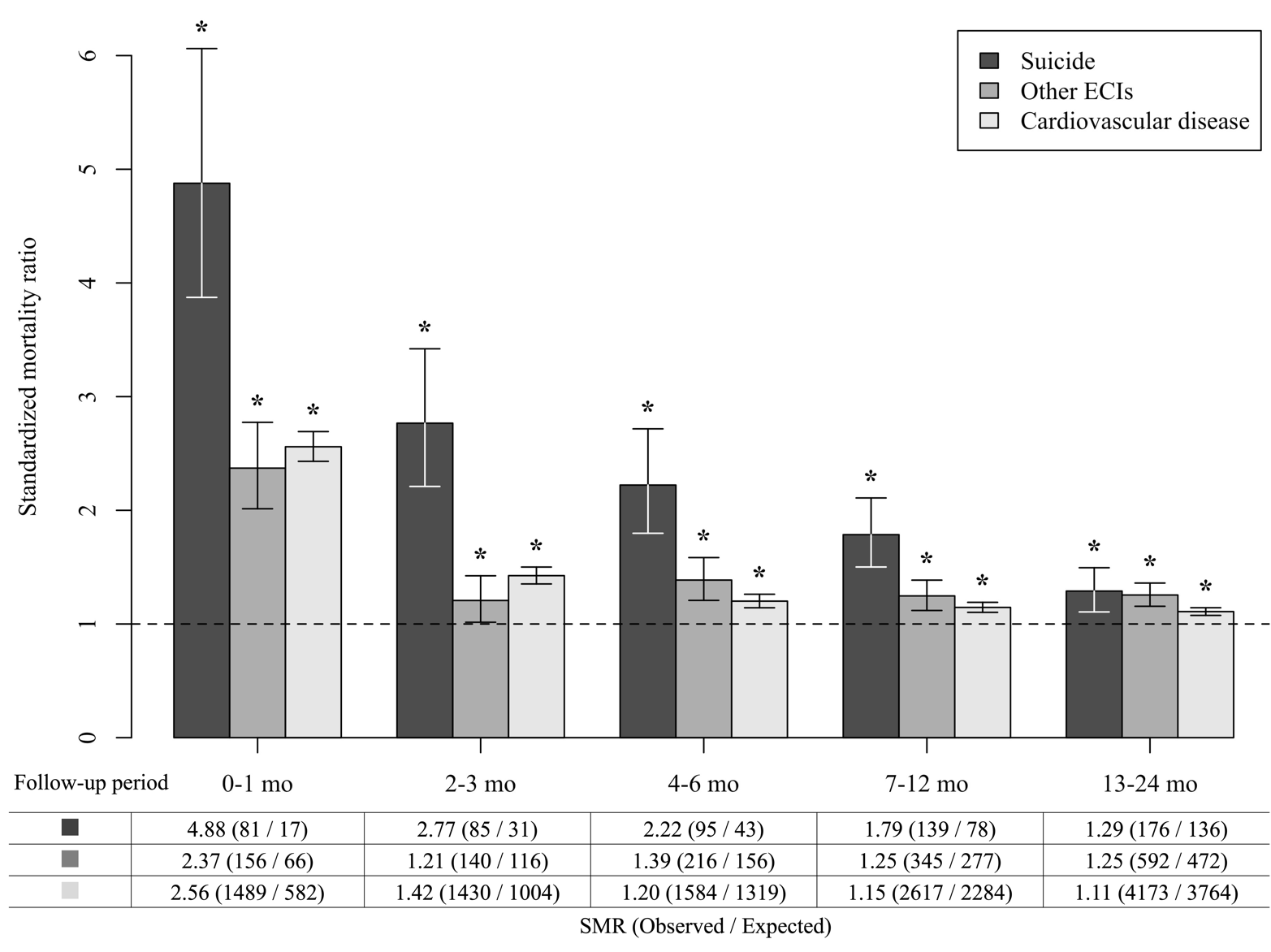


**Supplementary Table 1. Relative risks adjusted for all factors of interest, excluding patients with multiple primary tumors**

|  | **Relative risk (95% CI)** | | |
| --- | --- | --- | --- |
|  | **Suicide^†^** | **Other ECIs^†^** | **CVD^‡^** |
| Age (years) |  |  |  |
| 0–39 | 0.98 (0.54–1.79) | 0.31 (0.08–1.30) | 2.69 (1.60–4.53)^*^ |
| 40–49 | 1.44 (0.98–2.13) | 0.73 (0.37–1.46) | 1.33 (0.99–1.79) |
| 50–59 | 1.00 (Reference) | 1.00 (Reference) | 1.00 (Reference) |
| 60–69 | 1.20 (0.90–1.60) | 1.01 (0.72–1.40) | 0.99 (0.84–1.17) |
| 70–79 | 1.16 (0.87–1.54) | 0.89 (0.65–1.22) | 0.80 (0.68–0.94)^*^ |
| ≥80 | 0.83 (0.61–1.15) | 0.61 (0.45–0.83)^*^ | 0.52 (0.45–0.61)^*^ |
| Sex |  |  |  |
| Female | 1.09 (0.87–1.36) | 1.09 (0.96–1.24) | 1.17 (1.09–1.26)^*^ |
| Male | 1.00 (Reference) | 1.00 (Reference) | 1.00 (Reference) |
| Primary tumor site |  |  |  |
| Head and neck | 1.65 (1.00–2.72)^*^ | 1.30 (0.89–1.89) | 0.91 (0.75–1.12) |
| Esophagus | 2.25 (1.43–3.55)^*^ | 1.43 (0.99–2.07) | 1.08 (0.88–1.32) |
| Stomach | 1.21 (0.87–1.70) | 1.33 (1.08–1.63)^*^ | 1.14 (0.97–1.32) |
| Colon | 1.00 (Reference) | 1.00 (Reference) | 1.00 (Reference) |
| Rectum | 1.15 (0.77–1.72) | 0.82 (0.60–1.13) | 0.95 (0.80–1.12) |
| Liver and intrahepatic bile ducts | 1.32 (0.80–2.20) | 1.82 (1.38–2.40)^*^ | 1.39 (1.17–1.66)^*^ |
| Gallbladder and other biliary tract | 1.31 (0.68–2.49) | 1.38 (0.95–1.99) | 0.95 (0.77–1.16) |
| Pancreas | 1.55 (0.95–2.54) | 1.47 (1.04–2.08)^*^ | 1.04 (0.86–1.25) |
| Lung and bronchus | 1.01 (0.70–1.46) | 1.29 (1.03–1.61)^*^ | 1.07 (0.92–1.25) |
| Skin | 0.98 (0.53–1.83) | 1.58 (1.20–2.08)^*^ | 1.04 (0.85–1.28) |
| Breast | 1.18 (0.77–1.80) | 0.78 (0.56–1.09) | 0.69 (0.57–0.84)^*^ |
| Cervix uteri | 1.28 (0.66–2.46) | 1.59 (0.85–2.97) | 0.86 (0.63–1.16) |
| Corpus uteri | 0.99 (0.39–2.51) | 1.01 (0.47–2.16) | 0.82 (0.60–1.12) |
| Ovary | 1.10 (0.43–2.79) | 0.73 (0.27–1.97) | 0.77 (0.55–1.08) |
| Prostate | 0.67 (0.45–0.99)^*^ | 0.94 (0.75–1.19) | 0.63 (0.53–0.76)^*^ |
| Bladder | 1.57 (1.02–2.42)^*^ | 1.01 (0.75–1.36) | 0.98 (0.81–1.18) |
| Kidney and urinary organs | 0.81 (0.43–1.54) | 1.06 (0.72–1.56) | 0.99 (0.82–1.20) |
| Brain and other parts of the CNS | 0.83 (0.20–3.42) | 1.68 (0.74–3.80) | Excluded |
| Thyroid | 0.45 (0.16–1.24) | 0.59 (0.28–1.27) | 0.53 (0.40–0.71)^*^ |
| Malignant lymphoma | 1.04 (0.63–1.71) | 0.86 (0.59–1.26) | 0.81 (0.68–0.98)^*^ |
| Multiple myeloma | 0.96 (0.38–2.47) | 0.97 (0.55–1.71) | 0.81 (0.61–1.08) |
| Leukemia | 0.12 (0.02–0.90)^*^ | 2.02 (1.33–3.07)^*^ | 0.53 (0.39–0.72)^*^ |
| Other | 1.05 (0.69–1.59) | 1.81 (1.42–2.29)^*^ | 0.97 (0.82–1.15) |
| Extension of tumor |  |  |  |
| Localized | 1.00 (Reference) | 1.00 (Reference) | 1.00 (Reference) |
| Regional | 1.60 (1.28–1.99)^*^ | 1.33 (1.15–1.54)^*^ | 1.27 (1.16–1.40)^*^ |
| Metastatic | 2.50 (1.96–3.18)^*^ | 1.53 (1.28–1.82)^*^ | 1.59 (1.43–1.76)^*^ |
| Unknown/other | 2.14 (1.64–2.80)^*^ | 2.01 (1.73–2.34)^*^ | 2.50 (2.28–2.74)^*^ |

Abbreviations: CI, confidence interval; CNS, central nervous system; CVD, cardiovascular disease; ECI, externally caused injury; RR, relative risk; SMR, standardized mortality ratio

All variables were included in the multivariate model.

^†^ Relative risk was quantified using Poisson regression model.

^‡^ Relative risk was quantified using negative binomial regression model.

^*^ p <0.05
